# Supplementary material for: Identification, and Experimental and Bioinformatics Validation of an Immune-Related Prognosis Gene Signature for Low-Grade Glioma Based on mRNAsi
Source: Cancers (Basel). 2023 Jun 19;15(12):3238. doi: 10.3390/cancers15123238 (PMC10296549; doi:10.3390/cancers15123238)
Supplement: Supplementary file 1 [file cancers-15-03238-s001.zip › cancers-2362274-supplementary.pdf]

Supplementary Table S1

	Forward (5'-3' sequence)	Reverse (5'-3' sequence)
SLC11A1	CAAAGGCTAAGCGGGTCCAG	AGCAGCACCCAGAGAAGTTTG
C3AR1	GCAGGTTCCTATGCAAGCTC	CACATCACAAAAGCCACCAC
IL18	ATCGCTTCCTCTCGCAACAA	GTCCGGGGTGCATTATCTCT
MSR1	CAGTGCTGCTTTCTTTAGGACG	ACTTCAGGAGTTGAGCTGCC
